# Identification of Myocardial Disarray in Patients With Hypertrophic Cardiomyopathy and Ventricular Arrhythmias

**DOI:** 10.1016/j.jacc.2019.02.065

**Published:** 2019-05-28

**Authors:** Rina Ariga, Elizabeth M. Tunnicliffe, Sanjay G. Manohar, Masliza Mahmod, Betty Raman, Stefan K. Piechnik, Jane M. Francis, Matthew D. Robson, Stefan Neubauer, Hugh Watkins

**Affiliations:** a Division of Cardiovascular Medicine, Radcliffe Department of Medicine, University of Oxford, Oxford, United Kingdom; b Nuffield Department of Clinical Neurosciences, University of Oxford, Oxford, United Kingdom

**Keywords:** diffusion tensor cardiac magnetic resonance imaging, disarray, fractional anisotropy, hypertrophic cardiomyopathy, risk stratification, sudden cardiac death, ventricular arrhythmia, ADC, apparent diffusion coefficient, CMR, cardiac magnetic resonance, DT-CMR, diffusion tensor cardiac magnetic resonance, ECV, extracellular volume, FA, fractional anisotropy, HA, helix angle, HCM, hypertrophic cardiomyopathy, ICD, implantable cardioverter-defibrillator, LGE, late-gadolinium enhancement, LV, left ventricular, NSVT, nonsustained ventricular tachycardia, SCD, sudden cardiac death, SA, sheetlet-normal angle, SNR, signal-to-noise ratio

## Abstract

**Background:**

Myocardial disarray is a likely focus for fatal arrhythmia in hypertrophic cardiomyopathy (HCM). This microstructural abnormality can be inferred by mapping the preferential diffusion of water along cardiac muscle fibers using diffusion tensor cardiac magnetic resonance (DT-CMR) imaging. Fractional anisotropy (FA) quantifies directionality of diffusion in 3 dimensions. The authors hypothesized that FA would be reduced in HCM due to disarray and fibrosis that may represent the anatomic substrate for ventricular arrhythmia.

**Objectives:**

This study sought to assess FA as a noninvasive in vivo biomarker of HCM myoarchitecture and its association with ventricular arrhythmia.

**Methods:**

A total of 50 HCM patients (47 ± 15 years of age, 77% male) and 30 healthy control subjects (46 ± 16 years of age, 70% male) underwent DT-CMR in diastole, cine, late gadolinium enhancement (LGE), and extracellular volume (ECV) imaging at 3-T.

**Results:**

Diastolic FA was reduced in HCM compared with control subjects (0.49 ± 0.05 vs. 0.52 ± 0.03; p = 0.0005). Control subjects had a mid-wall ring of high FA. In HCM, this ring was disrupted by reduced FA, consistent with published histology demonstrating that disarray and fibrosis invade circumferentially aligned mid-wall myocytes. LGE and ECV were significant predictors of FA, in line with fibrosis contributing to low FA. Yet FA adjusted for LGE and ECV remained reduced in HCM (p = 0.028). FA in the hypertrophied segment was reduced in HCM patients with ventricular arrhythmia compared to patients without (n = 15; 0.41 ± 0.03 vs. 0.46 ± 0.06; p = 0.007). A decrease in FA of 0.05 increased odds of ventricular arrhythmia by 2.5 (95% confidence interval: 1.2 to 5.3; p = 0.015) in HCM and remained significant even after correcting for LGE, ECV, and wall thickness (p = 0.036).

**Conclusions:**

DT-CMR assessment of left ventricular myoarchitecture matched patterns reported previously on histology. Low diastolic FA in HCM was associated with ventricular arrhythmia and is likely to represent disarray after accounting for fibrosis. The authors propose that diastolic FA could be the first in vivo marker of disarray in HCM and a potential independent risk factor.

Predicting sudden cardiac death (SCD) in hypertrophic cardiomyopathy (HCM) remains a challenge because most patients have a normal life expectancy (1). Current clinical risk stratification guidelines lack the high sensitivity and specificity needed to accurately predict this devastating complication, because they are based on clinical features alone 2, 3. The hallmark feature specific to patients who die suddenly with HCM is extensive myocardial disarray 4, 5, and this is a likely focus for re-entrant ventricular arrhythmias leading to SCD (6). Measuring the extent of disarray could provide a better and more mechanistic marker of arrhythmic SCD, were disarray not solely a post mortem finding.

Diffusion tensor cardiac magnetic resonance (DT-CMR) is a novel imaging technique capable of visualizing myocardial microstructure by mapping the diffusion of water molecules. DT-CMR is technically challenging and acquisition times are prohibitively long due to low signal-to-noise ratio (SNR). Thus, DT-CMR has largely been used ex vivo. Extensive validation against histology has shown that the greatest diffusion occurs along the length of myocytes and demonstrates the classical counterclockwise rotation of helix angles (HA) from a left-handed helix in the epicardium, circumferential in the mid-wall, to a right-handed helix in the endocardium 7, 8, 9. Diffusion in the other 2 dimensions corresponds to the laminar organization of myocytes in sheetlets and sheetlet-normal directions (10). Recent advancements in pulse sequence design and magnetic resonance hardware have now made in vivo DT-CMR feasible and reproducible in humans 11, 12.

Diffusion occurs in 3 dimensions. The directionality of this 3-dimensional diffusion within an imaging voxel is quantified by a scalar measure called fractional anisotropy (FA), calculated from the diffusion tensor. FA of zero describes random diffusion (perfect isotropy), whereas FA of 1 describes diffusion in only a single linear direction (perfect anisotropy) (Figure 1). Thus, FA is expected to be high in voxels with coherently aligned myocytes. Conversely, FA is expected to be reduced in voxels with differing myocyte orientations and in HCM due to disarray and expanded extracellular space (Figure 2).

**Figure 1:**
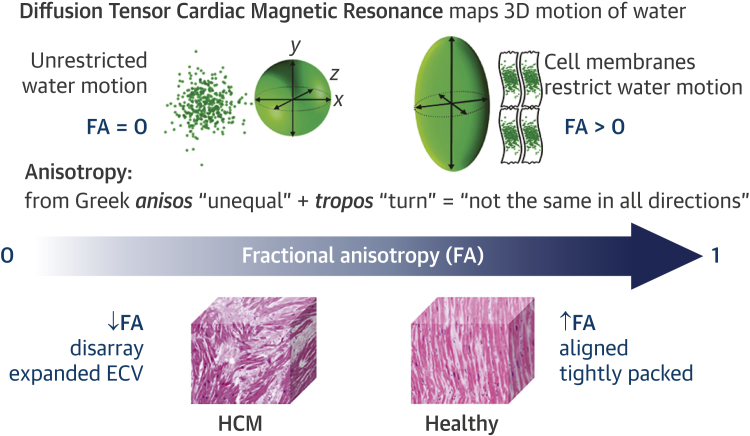
Diffusion Tensor Cardiac Magnetic Resonance Diffusion tensor cardiac magnetic resonance (DT-CMR) maps the diffusion of water molecules in 3 dimensions (3D). Fractional anisotropy (FA) calculated from the diffusion tensor quantifies the directionality of water diffusion within each imaging voxel (2.8 × 2.8 × 8 mm^3^) as its motion is impeded by several million myocytes and the surrounding interstitium. Without barriers, water motion is random and equal in all directions, which can be represented as a sphere using the diffusion tensor and has an FA of zero (perfect isotropy). Cell membranes act as barriers restricting water motion along the long axis of myocytes. Thus, FA is expected to be high in voxels with coherently aligned myocytes with a consistent orientation. Conversely, FA is expected to be low in voxels with differing myocyte orientations and in hypertrophic cardiomyopathy (HCM) due to disorganized cell orientations and expanded extracellular volume (ECV) (39).

**Figure 2:**
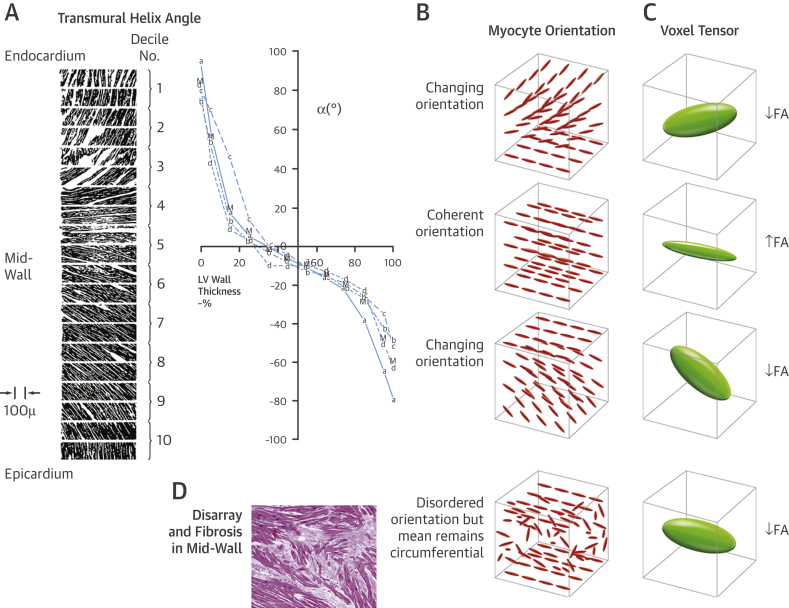
The Proposed Effect of Transmural Helical Myocyte Orientation on FA Streeter’s classic micrograph of transmural variation of helix angles from endocardium (0% wall thickness) to epicardium in a canine left ventricle (15)**(A)**. Approximately 3 DT-CMR imaging voxels (2.8 × 2.8 × 8 mm^3^) span the myocardium transmurally in diastole **(B)**. In the endo- and epicardial voxels, myocytes are progressively changing from a longitudinal to circumferential orientation and vice versa, respectively. There are no marked transmural differences in myocyte diameter or fibrous tissue in healthy myocardium (40). Therefore, the wide distribution of myocyte orientations in the endo- and epicardium will reduce FA. Conversely, the narrow distribution of orientations in the mid-wall where myocytes are consistently circumferentially orientated will elevate FA **(C)**. Myocyte disarray and fibrosis in the mid-wall will reduce FA, due to the wide distribution of disorganized myocyte orientations and expanded extracellular space (39), but the overall mean voxel helix angle remains in the circumferential orientation **(D)**. Thus, helix angle is expected to be normal despite abnormal FA. LV = left ventricular; other abbreviations as in Figure 1.

Post-mortem histology has shown that the left ventricular (LV) mid-wall contains a band of circumferentially orientated myocytes 13, 14, 15, which in HCM is infiltrated by disarray and fibrosis, typically at the interventricular junctions and the hypertrophied segments (Central Illustration panel E) (14). Deranged myocyte orientations within disarray are not randomly distributed, and the overall average mid-wall orientation remains circumferential (16). Hence, HA in HCM are likely to be normal despite disarray (Figure 2). However, FA is expected to be low because water molecules diffuse more isotropically within enlarged myocytes of bizarre shapes and orientations, and within areas of fibrosis. Fibrosis is known to decrease FA in dilated cardiomyopathy and correlates negatively with histological measurements of collagen, a major component of fibrosis (17). Therefore, low FA in HCM is likely to reflect a composite measure of both disarray and fibrosis. Late gadolinium enhancement (LGE) correlates with myocardial collagen in HCM, but not with disarray (18), and thus could potentially be used to separate the fibrosis component of FA to provide an in vivo measure of disarray. However, LGE only represents focal fibrosis rather than the concomitant diffuse fibrosis that is widespread in HCM. In the absence of edema and infiltration, extracellular volume (ECV) fraction is an excellent quantitative biomarker of diffuse fibrosis (19) but is yet to be histologically validated in HCM.

**Central Illustration:**
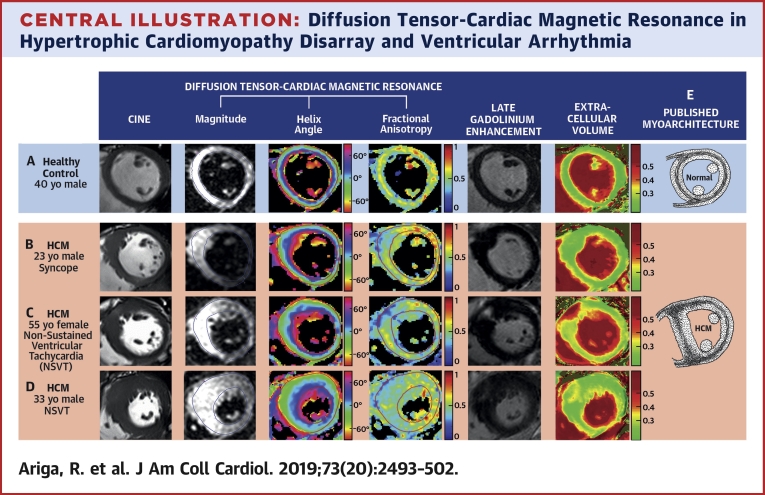
Diffusion Tensor-Cardiac Magnetic Resonance in Hypertrophic Cardiomyopathy Disarray and Ventricular Arrhythmia Multimodal imaging of left ventricular myoarchitecture and disarray using diffusion tensor-cardiac magnetic resonance (DT-CMR), late gadolinium enhancement (LGE), and extracellular volume (ECV) mapping. DT-CMR can provide in vivo assessment of left ventricular myoarchitecture. The helix angle (HA) is the average myocyte orientation, and fractional anisotropy (FA) is a surrogate measure of underlying cell organization. The mid-ventricular slice at diastole in healthy control subjects **(A)** and patients with hypertrophic cardiomyopathy (HCM) **(B–D)** demonstrated similar HA distributions, but marked differences in FA. There was an almost complete mid-wall ring of high FA **(yellow/orange)** in control subjects **(A)**, consistent with the classical description of circumferentially aligned mid-wall myocytes **(E)**, which was also present in the HA map. By contrast, this ring was disrupted by reduced FA **(B and C)** or was absent **(D)** in HCM. These patterns were consistent with previously published HCM histology that shows disarray and fibrosis invading the mid-wall at the insertion point and hypertrophied segments **(E)**(14). Low FA in the anteroseptum matched areas of focal LGE and elevated ECV **(C)** in keeping with fibrosis contributing to low FA. But low FA could not be explained by fibrosis in all cases. In some instances, low FA in the anteroseptum was present with no detectable LGE or elevated ECV **(B)**. Low FA also extended beyond areas of patchy LGE and elevated ECV, with no remnants of a mid-wall ring **(D)**. Thus, low diastolic FA is likely to represent disarray after accounting for fibrosis, which can, for the first time, be measured in vivo and noninvasively, providing a potentially independent marker for HCM risk stratification. NSVT = nonsustained ventricular tachycardia.

DT-CMR acquired with stimulated echo measures diffusion throughout the cardiac cycle. Although this allows adequate time to probe microstructure, it is sensitive to cardiac strain. However, FA is robust to strain when DT-CMR is acquired from diastole to diastole, unlike the other DT-CMR parameters such as apparent diffusion coefficient (ADC) (measures magnitude of diffusion) and sheetlet-normal angle (SA), which require strain correction (20). FA is also independent of orientation and therefore rotationally invariant, making it robust to difficulties in aligning hearts of varying shape. Thus, we sought only to assess diastolic FA as a noninvasive biomarker of HCM myocardial architecture. We hypothesized that low FA in HCM represents the combination of disarray and fibrosis. Both these histological abnormalities are increased in HCM patients who die suddenly 4, 21. Thus, we also hypothesized that low FA represents the anatomic substrate for electrical instability and therefore examined the relationship between low FA and ventricular arrhythmia in HCM patients.

## Methods

Detailed methods are described in the Online Appendix. The National Research Ethics Committee (REC ref 12/LO/1979) approved the study, and informed written consent was obtained from each participant. Fifty patients with HCM and 30 age- and sex-matched healthy control subjects were recruited prospectively to undergo DT-CMR at 3-T (TIM Trio, Siemens) using the same stimulated echo acquisition mode single-shot echo planar imaging sequence as our previous intercenter reproducibility work (11). A single mid-ventricular short-axis slice was acquired at the diastolic pause. Each acquisition was an 18-heartbeat breath-hold including reference image (b = 15 s/mm^2^) and 6 diffusion-encoding directions (b = 350 s/mm^2^, for heart rate of 60 beats/min), which was repeated to obtain a minimum of 8 averages. Post-processing was performed as previously described (Online Appendix) (11) by a single observer (R.A.) who was blinded to clinical data. FA, ADC, HA, SA, and SNR estimates were calculated voxel-wise (spatial resolution = 2.8 × 2.8 × 8 mm^3^, interpolated to 1.4 × 1.4 × 8 mm^3^), and all measures except HA were averaged across segment and slice. HA was reported as the transmural HA gradient from the linear fit.

Cine, LGE, and ECV imaging were also acquired in all participants (Online Appendix). All HCM patients underwent 24-h Holter electrocardiographic monitoring as part of routine clinical care. Ventricular arrhythmias detected in the past and during follow-up were included in the analysis and was defined as ≥3 consecutive ventricular beats of ≥120 beats/min. A linear mixed-effects model was fitted to examine the effects of segmental LGE and ECV on FA between HCM and control subjects. A logistic regression was used to predict the odds of a patient having ventricular arrhythmia from their FA value. LGE, ECV, and maximum end-diastolic wall thickness (measured on cine) were then added as covariates to the regression model to test whether FA was an independent predictor of ventricular arrhythmia (Online Appendix).

## Results

### Study population characteristics

The study population characteristics are summarized in Tables 1 and 2. Median follow-up was 4.4 ± 0.9 years. Fifteen patients (30%) had nonsustained ventricular tachycardia (NSVT) captured on 24-h Holter electrocardiographic monitoring. No patient had sustained ventricular tachycardia. Runs of NSVT ranged from 4 to 15 beats (median 8 beats) with rates from 125 to 200 beats/min (median 175 beats/min). Four patients (8%) received implantable cardioverter defibrillators (ICD) with no appropriate therapies reported at median follow-up of 4.1 ± 1.0 years since ICD implantation.

**Table 1:** Characteristics of HCM Patients and Healthy Control Subjects

	HCM Patients (n = 50)	Healthy Control Subjects (n = 30)	p Value
Age, yrs	47 ± 14	46 ± 16	0.70
Male	37 (74)	21 (70)	0.70
Heart rate, beats/min	57 ± 10	58 ± 11	0.75
LV end-diastolic volume, ml	154 ± 32	157 ± 37	0.78
LV ejection fraction, %	76 ± 5	68 ± 5	**<0.0001**
LV mass index, g/m^2^	74 ± 23	54 ± 12	**<0.0001**
Left atrial diameter, mm	39 ± 7	32 ± 6	**<0.0001**
Maximum LV wall thickness, mm	21 ± 4	11 ± 1	**<0.0001**
ECV	0.32 ± 0.04	0.28 ± 0.03	**0.001**
LGE present	22 (44)	0 (0)	**—**
LGE, % LV mass	5 ± 7	0	**—**

Values are mean ± SD or n (%). **Bold** p values are significant.

ECV = extracellular volume; HCM = hypertrophic cardiomyopathy; LGE = late gadolinium enhancement; LV = left ventricular.

**Table 2:** Clinical and Genetic Characteristics of HCM Patients

Clinical	
NYHA functional class	
I	38 (76)
II	10 (20)
III	2 (4)
IV	0 (0)
NSVT	15 (30)
Unexplained syncope	3 (6)
Family history of SCD	7 (14)
Massive LVH ≥30 mm	0 (0)
Abnormal exercise BP response	5 (10)
SCD risk factors	
0	23 (46)
1	24 (48)
2	3 (6)
3	0 (0)
ESC HCM Risk-SCD score	2.6 (1.2–8.7)
Genetic	
No pathogenic variant	20 (40)
MYBPC3	17 (34)
MYH7	12 (24)
Troponin I	1 (2)

Values are n (%) or median (range).

BP = blood pressure; ESC = European Society of Cardiology; HCM = hypertrophic cardiomyopathy; LVH = left ventricular hypertrophy; NSVT = nonsustained ventricular tachycardia; NYHA = New York Heart Association; MYBPC3 = myosin-binding protein C; MYH7 = beta-myosin heavy chain; SCD = sudden cardiac death.

### DT-CMR maps

The Central Illustration shows typical LV images and subsequent FA and HA maps obtained by DT-CMR at diastole with corresponding cine, LGE, and ECV maps. HA maps in both HCM and control subjects showed the expected transmural helical progression of myocytes from the epicardial left-handed helix to the endocardial right-handed helix. FA maps in control subjects demonstrated an almost complete mid-wall ring of high FA (Central Illustration panel A) compared with the epicardium and endocardium. This matched the ring of coherently aligned circumferentially orientated myocytes revealed on the HA map and in published histology (Central Illustration panel E) 13, 14, 15. By contrast, this mid-wall ring was disrupted in HCM at the anterior junction and the hypertrophied anteroseptum (Central Illustration panels B and C), and in some cases, the ring was absent (Central Illustration panel D), again consistent with patterns seen in published HCM histology (Central Illustration panel E) (14). Areas of profoundly low FA matched the regions of LGE and elevated ECV (Central Illustration panel C), in line with fibrosis contributing to low FA, but low FA could not be explained by fibrosis in all cases. In some instances, FA was low in patients without LGE or elevated ECV (Central Illustration panel B) and extended beyond areas of fibrosis indicating disarray (Central Illustration panel D) (17).

### DT-CMR parameters in HCM and control subjects

**Figure 3:**
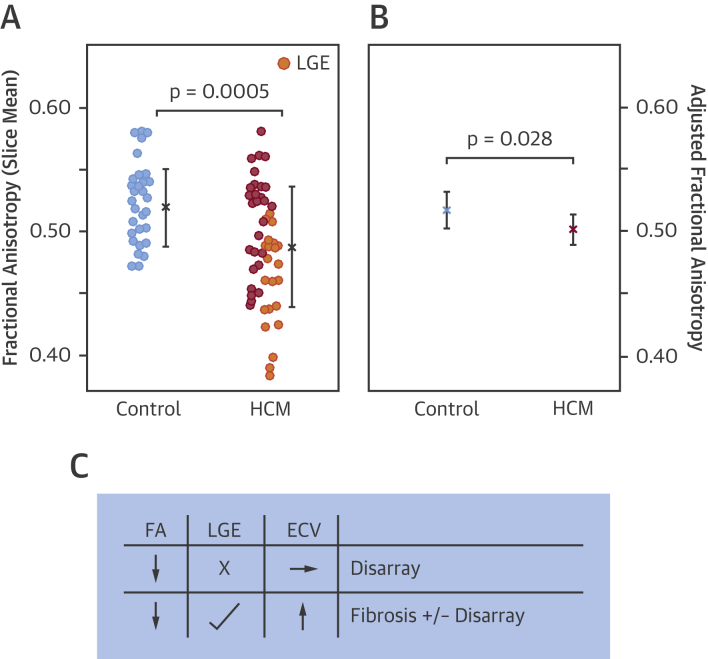
Reduced FA in HCM Even After Adjustment for Fibrosis Diastolic FA, averaged across the mid-ventricular slice, was reduced in HCM compared with healthy control subjects (**A**, slice mean ± SD). The wide standard deviation of FA in HCM demonstrates the heterogeneity within this disease population, and only a subset of patients had a lower FA than control subjects. HCM patients with late gadolinium enhancement (LGE) (n = 22; **orange**) had lower FA than those without LGE (p < 0.001). LGE and ECV, which are markers of fibrosis, were significant predictors of FA (p < 0.001). Yet FA adjusted for LGE and ECV remained reduced in HCM (**B**, linear mixed-effects model estimated marginal means with 95% confidence intervals), indicating that low FA may also demonstrate sensitivity to disarray. These results imply that fibrosis (presence of LGE and/or elevated ECV) contributes to low FA **(C)**. However, in the absence of fibrosis, low FA may instead represent disarray. Abbreviations as in Figure 1.

Overall diastolic FA, averaged across the slice, was reduced in HCM compared with control subjects (slice mean 0.49 ± 0.05 vs. 0.52 ± 0.03; p = 0.0005) (Table 3, Figure 3A). No group differences were detected in ADC, HA gradient, or SNR estimates. SA was reduced in HCM compared with control subjects in the segment of maximum thickness, that is, HCM diastolic sheetlet populations were radially, rather than longitudinally, orientated (circular mean 5 ± 23° vs. 84 ± 31°; p < 0.0001) (Table 3).

**Table 3:** DT-CMR Parameters in HCM Patients and Healthy Control Subjects

	HCM Patients (n = 50)	Healthy Control Subjects (n = 30)	p Value
FA	0.49 ± 0.05	0.52 ± 0.03	**0.0005**
ADC, ×10^−3^ mm^2^/s	1.29 ± 0.10	1.26 ± 0.12	0.20
HA gradient, degrees/mm	−7 ± 1	−8 ± 2	0.53
SA,°	5 ± 23	84 ± 31	**<0.0001**
SNR estimate	10.0 ± 2.5	10.4 ± 2.6	0.53

Values are slice mean ± SD, and for SA, circular mean ± SD. **Bold** p values are significant.

ADC = apparent diffusion coefficient; DT-CMR = diffusion tensor cardiac magnetic resonance; FA = fractional anisotropy; HA = helix angle; HCM = hypertrophic cardiomyopathy; SA = sheetlet-normal angle in segment of maximum thickness (0º denotes radial orientation of sheetlet populations); SNR = signal-to-noise ratio.

Although FA was globally reduced in HCM, this effect differed significantly across segments (interaction p < 0.0001). Minimum segmental FA corresponded to the maximally hypertrophied segment in every HCM patient. Within individuals, average segmental FA in HCM was reduced in the maximally hypertrophied segment (anterior, anteroseptum, or inferoseptum) compared to the inferolateral segment (0.44 ± 0.05 vs. 0.50 ± 0.08, difference 0.06 ± 0.08; p < 0.0001). By contrast, no significant difference was detected in control subjects between the segment with maximal wall thickness (anteroseptum or inferoseptum) compared to the inferolateral segment (0.51 ± 0.04 vs. 0.52 ± 0.07, difference 0.01 ± 0.07; p = 0.313).

### Relationship of FA with LGE and ECV

HCM patients with LGE (n = 22) had lower FA than those without LGE (slice mean 0.46 ± 0.04 vs. 0.51 ± 0.04; p = 0.00006) (Figure 3A). To investigate the relationship between FA and fibrosis, we examined per-segment average FA in a linear mixed-effects model with segmental LGE and ECV as covariates. The mixed model was fitted to account for any within-patient correlation or systematic variation across the 6 mid-ventricular segments. LGE and ECV were significant predictors of FA (segmental linear mixed-effects model, both p < 0.001), in keeping with fibrosis contributing to low FA. However, importantly, FA adjusted for LGE and ECV remained reduced in HCM (segmental linear mixed-effects model, estimated marginal means 0.50 [95% confidence interval (CI): 0.49 to 0.51] vs. 0.52 [95% CI: 0.50 to 0.53]; p = 0.028) (Figure 3B), suggestive that low FA may also be sensitive to disarray (Figure 3C).

### Relationship between FA and ventricular arrhythmia in HCM

**Figure 4:**
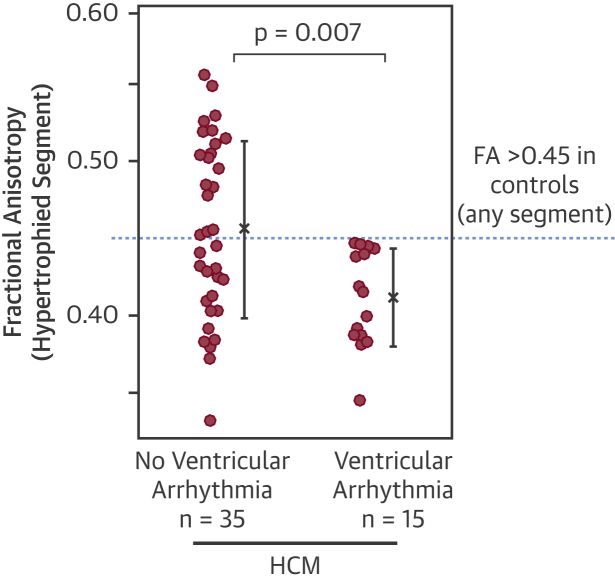
Reduced FA in HCM With Ventricular Arrhythmias Diastolic FA, measured in the hypertrophied segment, was reduced in HCM with ventricular arrhythmia compared with those without (mean ± SD). Some patients with no apparent ventricular arrhythmia also had low FA, perhaps in part reflecting the low sensitivity of a 1-day Holter. Interestingly, the lowest FA in any segment in any control subject was 0.45. Thus, there was no overlap in segmental FA values between control subjects and HCM patients with ventricular arrhythmia. Abbreviations as in Figure 1.

To investigate the relationship between FA and ventricular arrhythmia, we examined FA in the maximally hypertrophied segment as a potential substrate for arrhythmias. FA was reduced in HCM patients with documented ventricular arrhythmia compared with those without (n = 15; segmental mean 0.41 ± 0.03 vs. 0.46 ± 0.06; p = 0.007) (Figure 4). The lowest FA in any segment of any control subject was 0.45. Thus, there was no overlap in segmental FA values between control subjects and HCM patients with ventricular arrhythmia (Figure 4). There was only a trend toward increased fibrosis in HCM patients with ventricular arrhythmia compared to those without (LGE, 9 patients vs. 13; p = 0.14; ECV 0.33 ± 0.04 vs. 0.31 ± 0.04; p = 0.058) and no significant difference in hypertrophy (maximum LV wall thickness, 19 mm vs. 20 mm; p = 0.60), suggesting that disarray may be the driver for FA difference. No significant difference was detected in SA in HCM patients with and without ventricular arrhythmia (24 ± 33° vs. 0 ± 30°; p = 0.08), despite SA showing marked differences between HCM and control subjects.

To examine the relationships between FA, fibrosis, and maximum wall thickness in patients with and without ventricular arrhythmia, we used logistic regression. FA predicted the odds of a patient having ventricular arrhythmia. A decrease in FA of 0.05 increased the odds of ventricular arrhythmia by 2.5 (95% CI: 1.2 to 5.3; p = 0.015). This remained significant even after correcting for LGE, ECV, and maximum wall thickness by adding them as covariates to the regression model (p = 0.036).

## Discussion

In this paper, we show that DT-CMR provided in vivo visualization of normal and HCM myocardial architecture, which demonstrated high concordance with previously described post-mortem findings (14). Despite the relatively coarse spatial resolution of in vivo DT-CMR, which was on the scale of millimeters, diastolic FA was sensitive to microscopic structure and was able to image the well-documented circumferential alignment of myocytes in the mid-wall (13). FA was reduced in HCM compared with control subjects, but there was heterogeneity between HCM patients. Only 34% of patients demonstrated FA values below control subjects, some of whom had LGE. Using multimodal cardiac magnetic resonance (CMR), we were able to account for the contribution of fibrosis to FA. FA, adjusted for fibrosis, remained reduced in HCM, indicating that low FA may also demonstrate sensitivity to disarray. We showed that focal reduction of FA in the maximally hypertrophied segment of HCM patients was associated with ventricular arrhythmia. This association of low FA with ventricular arrhythmia persisted even after adjusting for fibrosis and hypertrophy, suggesting that adjusted FA could be measuring disarray and hence may provide a novel, independent risk factor.

### Concordance with other in vivo DT-CMR studies

Our quantitative DT-CMR parameters, ADC, FA, and HA in control subjects were comparable with previous intercenter reproducibility results using the same imaging protocol (11). Furthermore, we confirm abnormal diastolic SA in HCM as previously described (22). However, no significant SA differences were detected in HCM patients with and without ventricular arrhythmia, using our protocol. Our FA results in HCM are consistent with a previous, small study that also reported reduced FA in the septum compared with the free wall in 5 HCM patients using DT-CMR acquired at mid-systole (23). However, more recently, McGill et al. (12) found no difference in septal FA in 10 HCM patients when DT-CMR was acquired from end-systole to end-systole. This may in part be due to the small sample size. More than 10 patients within our cohort had no detectable difference in FA, which is likely to represent the significant proportion of HCM patients who have a benign clinical course with no adverse effect to life expectancy (24). Another factor is that FA measured using a stimulated echo diffusion sequence is affected by cardiac strain, which is most marked at peak systole (20). Although this effect can be corrected by acquiring accurate 3-dimensional strain data, it prolongs scan duration, increases data processing time, and increases the chance of systematic error arising (25). However, unlike systole, FA obtained at diastole is unchanged by strain correction, and therefore, our diastolic FA values have not needed adjustment for strain (20).

### Concordance with histology

Our FA maps in control subjects and HCM patients matched previous histological findings 13, 14, 15. The disruption of the mid-wall ring by low FA in HCM mirrored the findings of Kuribayashi and Roberts (14) in a study of 47 autopsied hearts with HCM. They reported destruction of the mid-wall circular orientation by disarray in 77% of patients at the insertion points, 33% at the septum, and 34% at the anterior and inferior walls.

Our HA maps in HCM showed similar transmural angular progression to control subjects, and there was no significant difference in HA between the 2 groups despite reduced FA in HCM. Whittaker et al. (16) found similar results in mid-wall septal sections. In control subjects, they found that myocyte orientation peaked sharply at the mean orientation angle of zero consistent with coherently aligned myocytes in a circumferential direction. However, in HCM, there was a wide, sometimes bimodal distribution of myocyte orientations, but the overall mean orientation angle still remained zero and circumferential. Thus, in HCM, the average HA within a voxel appeared to be normal, whereas FA, a measure of the overall variance of cell orientations, was abnormal (Figure 2).

### Detecting the anatomic substrate for arrhythmia in HCM

Previous studies have suggested that structural disruption such as fibrosis and disarray provide the conditions for re-entrant ventricular arrhythmias that lead to SCD (26). LGE has modest correlation to clinical risk factors for SCD (27), suggesting that other factors such as disarray may also be important (4). It is likely that there is a continuum within the HCM myocardium involving both disarray and fibrosis that increases vulnerability to arrhythmia. This could explain why HCM patients over 35 years of age are not risk-free (1), because fibrosis becomes the predominant post-mortem finding in this age group (4). Interestingly, none of the control subjects had FA <0.45 in any segment, which provided complete separation of control subjects from HCM patients with ventricular arrhythmia. This may represent a potential FA threshold for arrhythmic risk, if this normal limit persists in larger cohorts.

In our study, a proportion of patients without recorded ventricular arrhythmic events had similarly low FA values to those with ventricular arrhythmias. However, Holter monitoring may underestimate the true burden of ventricular arrhythmia (28). Continuous monitoring via ICD in HCM has shown a higher prevalence of NSVT than Holter monitoring (29). NSVT is a recognized marker of arrhythmic potential for precipitating SCD 30, 31, 32; however, most patients with NSVT do not die suddenly, suggesting heterogeneity within this group (33). The underlying structural substrate may be one source of this heterogeneity, and future studies will need to assess the relationship between extent, distribution, and location of disarray versus fibrosis with SCD or aborted SCD. In practice, using FA as a composite marker of disarray and fibrosis, rather than dissociating the fibrotic component, would be more convenient but may also be clinically useful as both these histological abnormalities are intrinsically linked to arrhythmic risk 4, 21.

### Study limitations

We were unable to study patients with severe breathlessness due to the repeated prolonged breath-holding requirements for DT-CMR. Participants had to provide consistency in the rate and depth of breath-holding to ensure the myocardium remained in an identical position during diffusion encoding. Furthermore, ectopy, which is often seen in HCM, prolonged scan times because optimal diffusion encoding is dependent on a regular RR interval. Shorter scan times are required before DT-CMR can be adopted for routine clinical use in symptomatic patients. This may be achievable with stronger CMR gradient systems and advanced DT-CMR sequences such as second-order motion-compensated spin echo 34, 35 and whole-heart methods (36). This also provides the potential to assess novel DT-CMR measures of disarray such as tractographic propagation angle (37) in HCM.

Our patients were relatively low risk because we could not include those with ICDs; thus, in this hypothesis-generating study, we could only test for association with predictors of risk rather than events. To document clinical utility of low FA for deciding whom to treat with ICD, we will need to measure its association with hard endpoints such as SCD, aborted SCD, and appropriate therapies from ICDs implanted after initial FA measurement. This will need to be tested prospectively in large-scale multicenter studies and registries such as the Hypertrophic Cardiomyopathy Registry (38).

## Conclusions

DT-CMR provided in vivo assessment of normal and HCM myocardial microstructure, which demonstrated concordance with histology. FA was reduced in HCM compared with control subjects. Low FA in HCM was associated with ventricular arrhythmia and is likely to represent disarray after accounting for fibrosis. We propose that diastolic FA could be the first in vivo marker of disarray in HCM and thus a potential independent risk factor for SCD.

### Acknowledgments

The authors thank Joanne Sellwood for nursing support, Rachel Given for data management, Dr. Elizabeth Ormondroyd and the Oxford Medical Genetics Laboratories for genetic data, Dr. Joanne Bates for the simulated diffusion images in Figure 1, and Dr. Lei Clifton for advice on statistics.

Perspectives **COMPETENCY IN MEDICAL KNOWLEDGE:** Disarray of cardiomyocytes is a hallmark feature of patients with HCM who experience sudden death. This histopathological substrate can be quantified noninvasively by diastolic FA calculated from DT-CMR imaging. Low diastolic FA is associated with an elevated risk of ventricular arrhythmia in patients with HCM.

**TRANSLATIONAL OUTLOOK:** Prospective studies are needed to assess the clinical utility of detecting FA by DT-CMR to stratify patients for risk of ventricular arrhythmias and guide selection of those who benefit from device-based therapy to prevent sudden death**.**
