# Supplementary material for: Identification of Myocardial Disarray in Patients With Hypertrophic Cardiomyopathy and Ventricular Arrhythmias
Source: J Am Coll Cardiol. 2019 May 28;73(20):2493–502. doi: 10.1016/j.jacc.2019.02.065 (PMC6548973; doi:10.1016/j.jacc.2019.02.065)
Supplement: Online Appendix [file mmc1.docx]

**Supplemental Material**

**1 Supplemental Methods**

**1.1 Study Population**

50 patients with established HCM were prospectively recruited from the University of Oxford Inherited Cardiac Conditions clinic, John Radcliffe Hospital, Oxford, UK. HCM patients were included if sarcomeric gene variants were deemed pathogenic with left ventricular hypertrophy (LVH) ≥13 mm or, in the absence of an identified mutation, if LVH ≥15 mm did not originate from another cause. The exclusion criteria were HCM phenocopies, coronary artery disease, diabetes, hypertension, body mass index (BMI) ≥35 kg/m^2^, atrial fibrillation and contraindications to CMR (including metallic devices). 30 age- and gender-matched healthy controls were non-smokers, BMI <34 kg/m^2^, without cardiovascular disease, hypertension, diabetes, or family history of cardiomyopathy or sudden cardiac death (SCD) and had a normal resting 12-lead ECG.

**1.2 Diffusion Tensor Cardiac Magnetic Resonance** **(DT-CMR)**

DT-CMR was performed at 3 Tesla (TIM Trio, Siemens) using an ECG-gated stimulated echo acquisition mode single-shot echo planar imaging sequence with monopolar diffusion encoding, as previously described (1-3). A single mid-ventricular short-axis slice was acquired during the diastolic pause. Each acquisition was an 18-heartbeat breath-hold including reference image (b=15 s/mm^2^) and 6 diffusion-encoding directions (b=350 s/mm^2^, for heart rate of 60 beats per minute), which was repeated to obtain a minimum of 8 averages. Images affected by breathing motion or ectopy were repeated. Sequence parameters were: fat saturation, TR = 2 RR intervals, TE = 22 ms, BW = 2442 Hz/pixel, GRAPPA parallel imaging acceleration factor of 2, FOV = 360 × 135 mm, acquisition matrix = 128 × 48 pixels, spatial resolution = 2.8 × 2.8 × 8 mm^3^, interpolated to 1.4 × 1.4 × 8 mm^3^. Total acquisition time was approximately 10 minutes for a single slice which consisted of a minimum of 8 breath-holds of 15-20 seconds duration with recovery time between breath-holds.

Post-processing was performed by a single observer (RA) who was blinded to clinical data, using custom-built software developed in house using MATLAB (MathWorks, MA, USA) as previously described (1), with the exception of calculating the diffusion tensor, which used Kingsley’s b-matrix method (4). Raw diffusion images were visually inspected, and motion-corrupted frames were rejected. A minimum of six averages per direction underwent rigid co-registration to correct for variation in cardiac position between breath-holds (5). Epicardial and endocardial region of interest (ROI), and anterior interventricular insertion point were defined on the reference diffusion image in conjunction with a corresponding cine image to improve recognition of trabeculae. Left ventricular endocardial trabeculae, right ventricular endocardium of the interventricular septum and papillary muscles were excluded from ROIs to avoid partial volume effects. The mid-ventricular slice was divided into six American Heart Association (AHA) segments for analysis using the anterior interventricular insertion point as reference.

Fractional anisotropy (FA), apparent diffusion coefficient (ADC), helix angle (HA), sheetlet-normal angle (SA) and signal-to-noise ratio (SNR) were calculated voxel-wise and all measures except HA were averaged across each segment and across the whole slice. HA was defined as the angle between the projection of the primary eigenvector onto the epicardial tangential plane and the short-axis plane. HA was reported as the transmural HA gradient from the linear fit. SA was defined as the angle between the projection of the tertiary eigenvector onto the longitudinal–radial plane and the short axis plane, and was positive for sheets rising towards the base from endocardium to epicardium, as previously described (6). Circular statistics were used to calculate voxel-wise SA (see Supplemental material 1.5). The set of low b-value reference images for each subject after rigid-body registration was used to produce SNR maps by dividing the mean value of each pixel the standard deviation of the signal within that pixel (7). Post-processing and analysis time was approximately 15 minutes per subject.

**1.3 Cardiovascular Magnetic Resonance (CMR) imaging**

All MR imaging was performed at 3 Tesla (TIM Trio, Siemens). Cine imaging was acquired using standard methods (8). LV volumes, function, mass and maximum segmental wall thickness at end-diastole were analyzed using cmr42© (Circle Cardiovascular Imaging, Calgary, Canada). T1 was acquired at the mid-ventricular short-axis slice using the Shortened MOdified Look-Locker Inversion recovery sequence (ShMOLLI) (9) before and after administration of gadolinium contrast. ECV was derived from blood hematocrit, pre- and post-contrast T1 calculated per slice and per segment; using custom-built software developed in house (MC-ROI, running on IDL v6.1) (10). Late gadolinium enhancement (LGE) imaging was performed in all subjects according to standard clinical protocols (11). LGE was defined as hyper-enhanced pixels with signal intensities of 5 standard deviations above the mean of normal myocardium in that slice.

**1.4 Clinical data collection**

All HCM patients underwent 24-hour Holter electrocardiographic (ECG) monitoring as part of routine clinical care. Ventricular arrhythmia both in the past and during follow-up were collated from the medical notes and defined as ≥3 consecutive ventricular beats of ≥120 beats per minute, in line with the definition used in HCM guidelines (12). Stored ECG data and appropriate device therapy was assessed for ventricular arrhythmia in patients with an implantable cardioverter defibrillator (n=4). The other conventional risk factors were noted: unexplained syncope, family history of SCD, massive LVH and abnormal exercise blood pressure response (13). HCM Risk-SCD score (14) was also calculated for each patient.

**1.5 Statistical analysis**

Normality was checked using the Shapiro-Wilk test. Data are expressed as mean ± standard deviation or median and range. Normally distributed data were compared using Student’s *t*-test and non-normally distributed data by Mann-Whitney U-test. Paired *t*-tests were used to compare the difference between septal and lateral segments within individuals. Categorical data were compared using Chi-square test. Circular statistics were used for SA since the angles “wrap” around a circle (i.e. SA of 90º is equivalent to -90º). This angular data was expressed as circular mean ± circular standard deviation and compared using Watson-Williams *F* test (15).

Segmental FA was analyzed using a linear mixed-effects model and expressed as estimated mean with 95% confidence intervals (16). The mixed model was fitted to account for any within-patient correlation or variation across the six mid-ventricular segments. Fixed effects included group (HCM *vs.* control) and segment. Individual subjects were included as random effect. FA was adjusted for fibrosis (which is expected to decrease FA (17,18)) by including segmental LGE (presence/absence) and segmental mean ECV as a covariate in the fixed effects part of the model. All model residuals were normally distributed without transformation of any of the variables. Univariate and multivariable binary logistic regression were used to assess FA as a predictor of ventricular arrhythmia in HCM. All tests were assumed to be statistically significant when p<0.05. Statistical analysis was performed with IBM SPSS Statistics, version 24.0 and STATA software version 14.2 (for linear mixed-effects model).

**REFERENCES**

1. Tunnicliffe E, Scott A, Ferreira P et al. Intercentre reproducibility of cardiac apparent diffusion coefficient and fractional anisotropy in healthy volunteers. J Cardiovasc Magn Reson 2014;16:31.

2. Reese TG, Weisskoff RM, Smith RN, Rosen BR, Dinsmore RE, Wedeen VJ. Imaging myocardial fiber architecture in vivo with magnetic resonance. Magn Reson Med 1995;34:786-91.

3. Nielles-Vallespin S, Mekkaoui C, Gatehouse P et al. In vivo diffusion tensor MRI of the human heart: Reproducibility of breath-hold and navigator-based approaches. Magn Reson Med 2013;70:454-465.

4. Kingsley PB. Introduction to diffusion tensor imaging mathematics: Part III. Tensor calculation, noise, simulations, and optimization. Concepts in Magnetic Resonance Part A 2006;28:155-179.

5. Guizar-Sicairos M, Thurman ST, Fienup JR. Efficient subpixel image registration algorithms. Optics letters 2008;33:156-158.

6. Hales PW, Schneider JE, Burton RA, Wright BJ, Bollensdorff C, Kohl P. Histo-anatomical structure of the living isolated rat heart in two contraction states assessed by diffusion tensor MRI. Prog Biophys Mol Biol 2012;110:319-330.

7. Sodickson DK, Griswold MA, Jakob PM, Edelman RR, Manning WJ. Signal‐to‐noise ratio and signal‐to‐noise efficiency in SMASH imaging. Magn Reson Med 1999;41:1009-1022.

8. Karamitsos TD, Hudsmith LE, Selvanayagam JB, Neubauer S, Francis JM. Operator induced variability in left ventricular measurements with cardiovascular magnetic resonance is improved after training. J Cardiovasc Magn Reson 2007;9:777-783.

9. Piechnik SK, Ferreira VM, Dall'Armellina E et al. Shortened Modified Look-Locker Inversion recovery (ShMOLLI) for clinical myocardial T1-mapping at 1.5 and 3 T within a 9 heartbeat breathhold. J Cardiovasc Magn Reson 2010;12:69.

10. White SK, Sado DM, Fontana M et al. T1 mapping for myocardial extracellular volume measurement by CMR: bolus only versus primed infusion technique. JACC Cardiovasc Imaging 2013;6:955-962.

11. Kellman P, Arai AE, McVeigh ER, Aletras AH. Phase‐sensitive inversion recovery for detecting myocardial infarction using gadolinium‐delayed hyperenhancement. Magn Reson Med 2002;47:372-383.

12. Authors/Task Force m, Elliott PM, Anastasakis A et al. 2014 ESC Guidelines on diagnosis and management of hypertrophic cardiomyopathy. The Task Force for the Diagnosis and Management of Hypertrophic Cardiomyopathy of the European Society of Cardiology (ESC). Eur Heart J 2014;35:2733-2779.

13. Gersh BJ, Maron BJ, Bonow RO et al. 2011 ACCF/AHA guideline for the diagnosis and treatment of hypertrophic cardiomyopathy: a report of the American College of Cardiology Foundation/American Heart Association Task Force on Practice Guidelines. J Thorac Cardiovasc Surg 2011;142:e153-e203.

14. O'Mahony C, Jichi F, Pavlou M et al. A novel clinical risk prediction model for sudden cardiac death in hypertrophic cardiomyopathy (HCM Risk-SCD). Eur Heart J 2014;35:2010-2020.

15. Berens P. CircStat: a MATLAB toolbox for circular statistics. J Stat Softw 2009;31:1-21.

16. Pinheiro J, Bates D. Mixed-Effects Models in S and S-Plus; Chambers J, Eddy W, Hardle W, Sheather S, Tierney L, editors. New York: Springer Verlag, 2000.

17. Bates J, McClymont D, Teh I, Kohl P, Schneider J, Grau V. Monte Carlo Simulations of Diffusion Weighted MRI in Myocardium: Validation and Sensitivity Analysis. IEEE Trans Med Imaging 2017.

18. Abdullah OM, Drakos SG, Diakos NA et al. Characterization of diffuse fibrosis in the failing human heart via diffusion tensor imaging and quantitative histological validation. NMR Biomed 2014;27:1378-1386.
